# One-Step Nucleic Acid Amplification (OSNA) of Sentinel Lymph Node in Early-Stage Endometrial Cancer: Spanish Multicenter Study (ENDO-OSNA)

**DOI:** 10.3390/cancers13174465

**Published:** 2021-09-04

**Authors:** María Dolores Diestro, Alberto Berjón, Ignacio Zapardiel, Laura Yébenes, Irune Ruiz, Arantza Lekuona, Marta Rezola, Ibon Jaunarena, Jaime Siegrist, Margarita Sánchez-Pastor, María Cuadra, Amaia Sagasta, Isabel Guerra, Luis I. Lete, Fernando Roldán, Carlo B. Marta, María J. Boillos, María J. Cardiel, Carlos López-de la Manzanara, Fernanda Relea, Pluvio J. Coronado, Alejandro Pascual, María J. Román, Gloria Peiró, Luis J. Matute, Beatriz Montero, Juan C. Muruzábal, Rosa Guarch, Cristina Zorrero, Ana Calatrava, Laia Ribot, Irmgard Costa, Alicia Hernández, David Hardisson

**Affiliations:** 1 Gynecologic Oncology Unit, Department of Gynecology and Obstetrics, Hospital Universitario La Paz, Hospital La Paz Institute for Health Research (IdiPAZ), Faculty of Medicine, Universidad Autónoma de Madrid, 28046 Madrid, Spain; ignacio.zapardiel@salud.madrid.org (I.Z.); jaime.siegrist@salud.madrid.org (J.S.); margarita.sanchezpastor@salud.madrid.org (M.S.-P.); ahernandezg@salud.madrid.org (A.H.); 2 Department of Pathology, Hospital Universitario La Paz, Molecular Pathology and Therapeutic Targets Group, Hospital La Paz Institute for Health Research (IdiPAZ), Center for Biomedical Research in the Cancer Network (Centro de Investigación Biomédica en Red de Cáncer, CIBERONC), Instituto de Salud Carlos III, Faculty of Medicine, Universidad Autónoma de Madrid, 28046 Madrid, Spain; alberto.berjon@salud.madrid.org (A.B.); laura.yebenes@salud.madrid.org (L.Y.); 3 Department of Pathology, Hospital Universitario Donostia Osakidetza, 20014 Donostia, Spain; irune.ruizdiaz@osakidetza.net (I.R.); marta.rezolabajineta@osakidetza.eus (M.R.); 4 Department of Gynecology, Hospital Universitario Donostia Osakidetza, 20014 Donostia, Spain; arantxa.lekuonaartola@osakidetza.net (A.L.); ibon.jaunarenamarin@osakidetza.eus (I.J.); 5 Department of Gynecology, Instituto de Investigación Bioaraba, OSI Araba Hospital Universitario, 01009 Vitoria-Gasteiz, Spain; maria.cuadracestafe@osakidetza.eus (M.C.); luisignacio.letelasa@osakidetza.eus (L.I.L.); 6 Department of Pathology, Instituto de Investigación Bioaraba, OSI Araba Hospital Universitario, 01009 Vitoria-Gasteiz, Spain; amaia.sagastalacalle@osakidetza.eus (A.S.); isabel.guerramerino@osakidetza.eus (I.G.); 7 Department of Gynecology, Hospital Clínico Universitario Lozano Blesa, 50009 Zaragoza, Spain; froldan@salud.aragon.es (F.R.); mjboillos@salud.aragon.es (M.J.B.); 8 Department of Pathology, Hospital Clínico Universitario Lozano Blesa, 50009 Zaragoza, Spain; cbmarta@salud.aragon.es (C.B.M.); mjcardiel@salud.aragon.es (M.J.C.); 9 Department of Gynecology, Hospital General Universitario Ciudad Real, Universidad de Castilla-La Mancha, 13005 Ciudad Real, Spain; calopez@sescam.jccm.es; 10 Department of Pathology, Hospital General Universitario Ciudad Real, Universidad de Castilla-La Mancha, 13005 Ciudad Real, Spain; fernandar@sescam.jccm.es; 11 Women Health Institute, Hospital Clínico Universitario San Carlos, IdISSC, Complutense University, 28040 Madrid, Spain; pluviojesus.coronado@salud.madrid.org; 12 Department of Pathology, Hospital Clínico Universitario San Carlos, 28040 Madrid, Spain; alejandro.pascual@salud.madrid.org; 13 Department of Gynecology, Hospital General Universitario Alicante and Alicante Institute for Health and Biomedical Research (ISABIAL), 03010 Alicante, Spain; roman_marsan@gva.es; 14 Department of Pathology, Hospital General Universitario Alicante and Alicante Institute for Health and Biomedical Research (ISABIAL), 03010 Alicante, Spain; peiro_glo@gva.es; 15 Department of Gynecology, Hospital Universitario y Politécnico La Fe, 46026 Valencia, Spain; matute_lui@gva.es; 16 Department of Pathology, Hospital Universitario y Politécnico La Fe, 46026 Valencia, Spain; montero_bea@gva.es; 17 Department of Gynecology, Complejo Hospitalario Universitario Navarra, 31008 Pamplona, Spain; jmuruzat@navarra.es; 18 Department of Pathology, Complejo Hospitalario Universitario Navarra, 31008 Pamplona, Spain; rosa.guarch.troyas@navarra.es; 19 Department of Gynecology, Hospital Fundación IVO, 46009 Valencia, Spain; czorrero@fivo.org; 20 Department of Pathology, Hospital Fundación IVO, 46009 Valencia, Spain; acalatrava@fivo.org; 21 Department of Gynecology, Corporación Sanitaria Parc Taulí Sabadell, 08208 Barcelona, Spain; jantoni@tauli.cat; 22 Department of Pathology, Corporación Sanitaria Parc Taulí Sabadell, 08208 Barcelona, Spain; icosta@tauli.cat

**Keywords:** endometrial cancer, sentinel lymph node, micrometastases, ultrastaging, one-step nucleic acid amplification, OSNA, cytokeratin 19

## Abstract

**Simple Summary:**

One-step nucleic acid amplification (OSNA) is an automated molecular diagnostic assay used to detect metastases by analyzing the levels of cytokeratin 19 mRNA in whole lymph nodes. It has been validated as an accurate and reliable tool for staging in several types of cancers and is included in the National Institute for Health and Care Excellence guidelines for the management of breast cancer. ENDO-OSNA is a large, observational, multicenter study designed to evaluate the efficacy of OSNA for the detection of sentinel lymph node (SLN) metastasis in patients with early-stage endometrial cancer. We found that the OSNA assay shows higher sensitivity, specificity, and diagnostic accuracy in the detection of SLN metastasis, including low-volume metastasis, compared to standard pathological ultrastaging. Moreover, OSNA could aid in the identification of patients with intermediate or high-risk endometrial cancer, and lead to treatment decisions that could improve their prognosis.

**Abstract:**

The objective of this study was to evaluate the efficacy of one-step nucleic acid amplification (OSNA) for the detection of sentinel lymph node (SLN) metastasis compared to standard pathological ultrastaging in patients with early-stage endometrial cancer (EC). A total of 526 SLNs from 191 patients with EC were included in the study, and 379 SLNs (147 patients) were evaluated by both methods, OSNA and standard pathological ultrastaging. The central 1 mm portion of each lymph node was subjected to semi-serial sectioning at 200 μm intervals and examined by hematoxylin–eosin and immunohistochemistry with CK19; the remaining tissue was analyzed by OSNA for CK19 mRNA. The OSNA assay detected metastases in 19.7% of patients (14.9% micrometastasis and 4.8% macrometastasis), whereas pathological ultrastaging detected metastasis in 8.8% of patients (3.4% micrometastasis and 5.4% macrometastasis). Using the established cut-off value for detecting SLN metastasis by OSNA in EC (250 copies/μL), the sensitivity of the OSNA assay was 92%, specificity was 82%, diagnostic accuracy was 83%, and the negative predictive value was 99%. Discordant results between both methods were recorded in 20 patients (13.6%). OSNA resulted in an upstaging in 12 patients (8.2%). OSNA could aid in the identification of patients requiring adjuvant treatment at the time of diagnosis.

## 1. Introduction

Endometrial cancer (EC) is the most frequently diagnosed gynecologic malignancy in Europe, with an estimated incidence of 130,051 new cases and 29,963 deaths in 2020 [1,2]. Most cases are low-grade, early-stage tumors, and are classified into low- or intermediate-risk categories, according to standard clinico-pathological features [3]. Surgery is the primary treatment, followed by adjuvant radiotherapy and/or chemotherapy depending on risk group stratification, and staging is based on pathological evaluation after surgery. Overall, the prognosis is favorable, with a 5-year survival of over 90% [4].

Around 10% of early-stage EC patients have lymph node metastases at the time of diagnosis and their presence negatively influences survival [5]. The prognostic value of systematic lymphadenectomy for patients with early-stage EC is controversial, with several studies reporting conflicting results [6,7,8,9,10,11]. Treatment of pelvic lymph nodes may not confer a direct therapeutic benefit and could increase the risk of complications such as lymphedema [9].

In recent years, sentinel lymph node biopsy (SLNB) has been incorporated in the management of early-stage EC as an alternative to systematic pelvic and para-aortic lymphadenectomy, and a negative SLN is widely accepted to confirm stage pN0 [12]. This procedure is being increasingly implemented in clinical practice [13,14,15]. The latest ESGO/ESTRO/ESP guidelines for the management of EC recommends SLNB for staging purposes in patients with low-risk/intermediate-risk tumors, although it can be omitted in patients without myometrial invasion. Therefore, SLNB is an acceptable alternative to systematic lymphadenectomy for lymph node staging in patients with FIGO stage I–II disease [3].

Moreover, SLNB reduces surgical staging morbidity associated with lymphadenectomy while maintaining valid prognostic information in early-stage EC [16]. SLNs are analyzed by standard pathological ultrastaging, which increases the sensitivity in the detection of micrometastases and isolated tumor cells (ITC) in the lymph node. The presence of micrometastases is associated with worse prognosis in patients who do not receive adjuvant treatment [17], whereas the presence of ITC, which should be staged as pN0(i+), does not have a clear impact on the outcome of EC [18,19].

Pathological ultrastaging is based on serial sectioning of the SLN with a combination of hematoxylin–eosin (H&E) and immunohistochemical (IHC) staining for cytokeratins. Compared to conventional histology, pathological ultrastaging can increase the detection rate of micrometastases by up to 20% [20]. Multiple protocols for SLN ultrastaging in EC have been described; however, no standardized routine protocol has been validated to date [21]. Moreover, pathological ultrastaging of SLNs is cost—and time—intensive [22], and is not suited for rapid, intraoperative diagnoses [20].

In this context, several molecular methods to improve the detection of metastasis in SLNs have been developed. The one-step nucleic acid amplification (OSNA) assay is a molecular-based metastasis detection system, which uses a reverse transcription loop-mediated isothermal amplification (RT-LAMP) assay to determine the number of mRNA copies of cytokeratin 19 (CK19) in the SLN [23]. OSNA assay accuracy for the detection of SLNB metastases has already been proven in several cancers, such as breast, lung, colorectal, prostate, cervical, and gastric carcinoma [24,25,26,27,28,29]. Recent studies have also demonstrated the applicability of the OSNA assay in the study of SLNs in EC [20,30,31,32,33,34]. A recent meta-analysis showed OSNA to be a highly accurate assay for the assessment of SLNs in EC [35]. OSNA has recently received CE marking for the detection of lymph node metastases in endometrial and cervical cancer, allowing its use in routine practice.

The aim of this large prospective, observational, and multicenter study (ENDO-OSNA) was to compare the efficacy of the OSNA method with standard pathological ultrastaging for detecting SLN metastasis in patients with early-stage EC and validate its clinical usefulness.

## 2. Materials and Methods

### 2.1. Study Design

ENDO-OSNA is a prospective, observational multicenter study including 11 Spanish hospitals (participating hospitals are listed in the Acknowledgements section). The primary objective of this study was to validate the performance of the OSNA assay in the evaluation of SLNs in early-stage EC. The performance of the OSNA assay was compared with pathological ultrastaging (multilevel sectioning with H&E and IHC for CK19, as described below). Pathological ultrastaging was considered the reference standard method (“gold standard”) for assessing the sensitivity, specificity, and diagnostic accuracy of the OSNA assay.

The study was approved by the local Ethics Committee of Hospital Universitario La Paz (code HULP: PI-1460) and was conducted in accordance with Good Clinical Practice (GCP) guidelines and the Declaration of Helsinki of the World Medical Association. All patients gave their written informed consent for inclusion in the study.

### 2.2. Patient Selection and Data Collection

This study involved 191 patients with early-stage EC. Patients fulfilling the following criteria were eligible for inclusion in the study: pathological confirmation of primary EC, pre-operative FIGO stage I or II, and laparoscopy surgical staging by hysterectomy with bilateral salpingo-oophorectomy with SLN mapping. The study was performed between January 2016 and January 2018. Data on age, histological type of tumor, histological grade, presence or absence of lymphovascular space invasion (LVSI), tumor size, myometrial invasion, and result of the pelvic or para-aortic lymphadenectomy (when performed), SLN size and weight, were collected for all patients. The histological type of the EC was established according to the 2014 World Health Organization classification of tumors of the endometrium [36]. Clinico-pathological features, OSNA, and pathological ultrastaging results were retrieved from the pathological reports and the patients’ clinical records. Data were collected anonymized in an Excel spreadsheet and Access database (Microsoft, Redmond, WA, USA, version 2016). All data obtained from the study were considered confidential and treated in accordance with the Spanish Law on the Protection of Personal Data.

### 2.3. SLN Biopsy and Processing

After SLN mapping by a combination of radioisotope tracer and/or methylene blue/green indocyanine, SLNs were processed as previously described [31]. Briefly, SLNs were dissected away from the surrounding fat, measured (max. diameter, mm) and weighed. Nodes that weighed <40 mg were excluded and were processed following only the pathological ultrastaging protocol. In nodes weighing >40 mg and measuring >3 mm along the largest dimension, a central longitudinal 1 mm slice was immediately taken from each node using a fresh scalpel. The remaining tissue was frozen and stored at −80 °C until the OSNA assay was performed. The central slice was fixed in formalin and embedded in paraffin for pathological ultrastaging. This section was sliced at 200 µm intervals for a total of four slides per block. At each level, two sections of 4 µm were taken: one section was examined with H&E and the other section was processed for IHC using anti-CK19 monoclonal antibody. The size of node metastases was estimated with an eyepiece micrometer. The following criteria were applied: macrometastases measured >2 mm; micrometastases were defined as a focus of metastasis measuring >0.2 mm and ≤2 mm. Isolated tumor cells were defined as microscopic clusters and single cells measuring 0.2 mm or less and were considered as negative for metastasis (pN0i+). The remaining tissue of each SLN was analyzed following the OSNA assay protocol.

### 2.4. OSNA Assay

OSNA was performed according to the manufacturer’s instructions [23]. The lymph node tissue was homogenized in 4 mL of lysis buffer (Lynorhag, Sysmex, Kobe, Japan) for 90 s and centrifuged for 1 min at 10,000× *g* at room temperature. Then, CK19 mRNA was amplified by RT-LAMP with a ready-to-use reagent kit (Lynoamp, Sysmex) in an RD-100i OSNA system. A standard positive control sample containing 5000 copies of CK19 mRNA/µL and a negative control sample containing 0 copies/µL were used for calibration in each study. The results were expressed as the level of CK19 mRNA. Based on the cutoff values determined by Nagai et al. [30] for the OSNA assay in EC tissues, values <250 copies/µL of CK19 mRNA were considered negative (−); values between 250 and 4999 copies/µL were considered micrometastases (+); and values ≥5000 copies/µL were considered macrometastases (++).

### 2.5. Statistical Analysis

The descriptive statistics of the analyzed variables included the measures of central tendency and dispersion for the quantitative variables, and the absolute and relative frequencies for the qualitative variables, with a 95% confidence interval in both cases. Quantitative results were expressed as mean ± standard deviation.

The discriminative capacity of the OSNA assay in the detection of SLN metastases in EC was evaluated by means of the ROC (receiver operating characteristic) curve between the different values observed in the number of CK19 mRNA copies/µL and the reference results (negative/positive) obtained by the pathological ultrastaging.

The diagnostic value of the OSNA assay was evaluated by calculating sensitivity, specificity, positive predictive value (PPV), negative predictive value (NPV), Kappa index, and diagnostic accuracy considering the OSNA assay as the diagnostic test value, and the result of the pathological ultrastaging as the reference standard method in the detection of SLN metastases at the patient level. The diagnostic accuracy was assessed by the percentage of discordance, sensitivity (%), specificity (%), false positives (%), false negatives (%), negative predictive value (NPV) (%), and Kappa index. The McNemar test was also applied to assess whether the percentage of false positives was higher than that of false negatives. Data were analyzed using the SAS statistical software, version 9.4 (SAS Institute Inc., Cary, NC, USA). Differences were considered significant with *p* values < 0.05.

## 3. Results

### 3.1. Patient Characteristics

A total of 191 patients with early-stage EC were included in the study. The median age was 62 years (range 34–85). Final tumor histological classification included 158 endometrioid carcinomas (82.7%), 16 serous carcinomas (8.4%), 5 carcinosarcomas (2.6%), 5 clear cell carcinomas (2.6%), 3 mixed carcinomas (1.6%) and 2 mucinous carcinomas (1%). The remaining 2 tumors (1%) were of other types. Tumors of histological grades 1, 2, and 3 were found in 85 (44.5%), 59 (30.9%), and 41 (21.5%) patients, respectively. LVSI was found in 61 (32.1%) cases. One hundred and eighteen patients (61.8%) underwent pelvic lymphadenectomy and 62 (32.5%) underwent para-aortic lymphadenectomy. The demographic data and baseline characteristics, as well as pathological features, are summarized in Table 1.

### 3.2. Sentinel Lymph Nodes Characteristics

In total, 526 SLNs were obtained from 191 patients. A median of 2 SLNs (range 1–10) were analyzed per patient. Table 2 summarizes the characteristics of the SLNs included in the study. Overall, OSNA detected 19.7% of metastases in the patients analyzed, of which 14.9% corresponded to micrometastasis and 4.8% to macrometastasis. On the other hand, pathological ultrastaging detected 8.8% of metastases in our series, of which 3.4% corresponded to micrometastasis and 5.4% to macrometastasis.

### 3.3. Comparison of the OSNA Assay with Pathological Ultrastaging

SLNs obtained from 147 patients with EC were used to compare the sensitivity (y-axis) and specificity (x-axis) of OSNA with pathological ultrastaging. Receiver operating characteristic (ROC) curve evaluation showed that OSNA had good discriminative power compared to ultrastaging and was highly effective in detecting SLN metastases in terms of the area under the ROC [AUROC 0.91 (95% CI, 0.82–1.0)].

**Figure 1:**
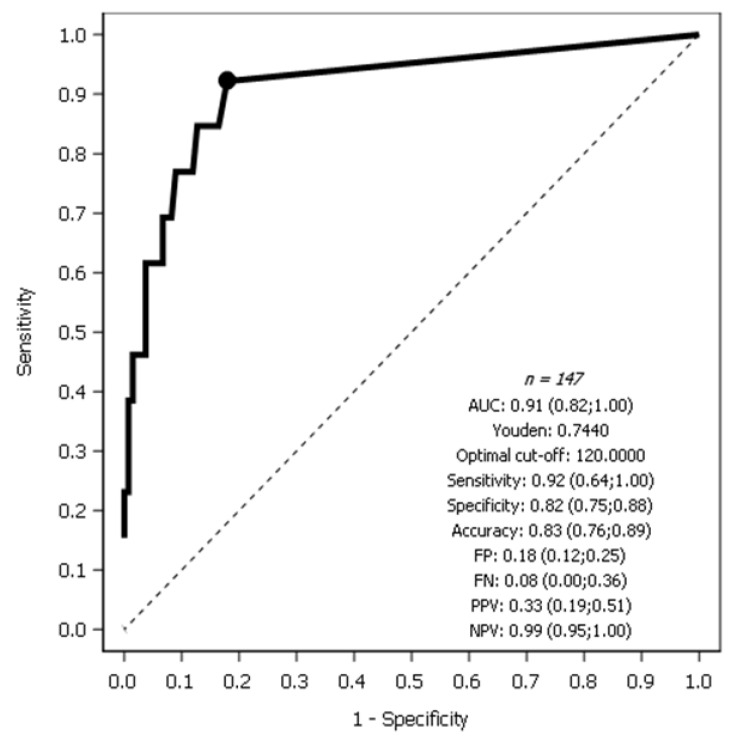
ROC curve showing the OSNA assay vs. pathological ultrastaging for the detection of SLN metastasis in endometrial cancer.

The sensitivity of the OSNA assay was 92%, specificity was 82%, and diagnostic accuracy was 83%. NPV was 0.99 (0.95; 1.00) (Figure 1). This high sensitivity, high specificity, and high NPV demonstrated the efficacy and sensitivity of the OSNA technique compared to the standard ultrastaging technique for the detection of metastases in SLNs in EC.

### 3.4. Consistency between Pathological Ultrastaging and OSNA

The consistency between the OSNA assay and pathological ultrastaging is shown in Table 3. Overall, more metastases were detected by the OSNA technique than by the standard pathological ultrastaging protocol (*p* < 0.001).

Of the 147 patients studied, 20 (13.6%) had discordant results between OSNA and pathological ultrastaging: 18 had SLNs that were histologically negative but positive in the OSNA assay (false-positive cases), and 2 were positive by pathological ultrastaging but negative in the OSNA assay (false-negative cases). Table 4 summarizes the discordant results between the OSNA assay and pathological ultrastaging.

In 16 of the 18 cases that had histologically negative SLNs, micrometastases were identified by OSNA (<5000 CK19 mRNA copies). In the remaining two cases, macrometastases were detected by OSNA (>5000 CK19 mRNA copies/µL).

OSNA also resulted in two false-negative results in which the number of copies/µL of CK19 mRNA detected was too low to be deemed positive (0 and 120 CK19 mRNA copies/µL, respectively), yet they were categorized as micrometatases by pathological ultrastaging. One of these cases showed very low expression of CK19 mRNA copies, indicating the presence of ITCs.

### 3.5. Consistency between TNM and FIGO Staging by OSNA and Ultrastaging

Interestingly, in 12 discordant cases, the results from the OSNA assay upstaged patients’ tumor from FIGO IA to FIGO IIIC1 (*n* = 7), FIGO IB to FIGO IIIC1 (*n* = 4), and FIGO II to FIGO IIIC1 (*n* = 1). Overall, OSNA resulted in an upstaging of 8.16% (12/147) of patients (Table 5).

LVSI, lymphovascular space invasion.

Notably, no metastases were found in non-SLNs from patients with OSNA-negative/ultrastaging-positive results that underwent lymphadenectomy. On the other hand, in 3 out of 19 (15.8%) cases with OSNA-positive/ultrastaging-negative results, lymphadenectomy revealed metastases in non-SLNs (Table 6). Together, these findings highlight the usefulness of OSNA in cancer staging and guiding treatment decisions.

## 4. Discussion

In this large, prospective, observational, multicenter study (ENDO-OSNA), we analyzed the usefulness of the OSNA assay compared to conventional pathological ultrastaging (serial H&E + IHC) in detecting SLN metastasis in a series of 191 patients with early-stage EC. In line with previous results by our group and others [20,30,31,32], OSNA showed high sensitivity (92%), specificity (82%), high NPV (99%), and diagnostic accuracy (83%) for the detection of SLN metastasis in EC. Compared to pathological ultrastaging, OSNA was able to detect 10.9% more metastasis-positive patients. Furthermore, the OSNA method upstaged 12 of 147 patients (8.16%) in our study. Eleven patients, who were staged as FIGO I according to pathological ultrastaging, were in fact FIGO stage IIIC by OSNA. This has important clinical implications, since FIGO stage I is considered either low-risk (IA) or intermediate-risk (IB), whereas FIGO stage IIIC is considered high-risk [3] and should be managed with adjuvant treatment. In this sense, it has been demonstrated that adjuvant treatment can improve outcomes for women with high-risk EC [37], particularly if chemoradiotherapy is used rather than radiotherapy alone [38]. Failure to detect low-volume lymph node metastases by conventional pathologic ultrastaging is considered a possible explanation for the 10–15% of node-negative endometrial carcinoma patients who experience disease relapse within 5 years of surgery. Therefore, detecting SLN metastasis at the time of initial diagnosis and surgical treatment could be useful to ensure that these patients receive early adjuvant therapy, potentially improving their prognosis and survival [39,40,41,42]. In this context, OSNA could help identifying those patients who could benefit from adjuvant therapy. Moreover, OSNA offers several advantages compared to pathological ultrastaging: it is faster, provides highly specific and reproducible results, and analyzes the whole lymph node, minimizing the risk of sampling bias that may arise as a result of analyzing just a few tissue sections, as occurs with pathological ultrastaging. The OSNA assay has already been validated for other types of tumors and is recommended in several guidelines for the management of breast (NICE) [43] and colorectal cancer (Spanish Society of Medical Oncology and the Spanish Society of Pathology) [44].

A total of 20 discordant cases (13.6%) were observed between both techniques, including two cases of macrometastases detected by OSNA with >5000 CK19 mRNA copies/µL. There were 18 cases in which the result of pathological ultrastaging was negative, but the OSNA result was positive (false-positive results, considering pathological ultrastaging as the reference method for the analysis of SLN). In 16 of those cases, OSNA detected low CK19 mRNA copy numbers, indicative of low-volume metastases (micrometastases) that were not detected by pathological ultrastaging.

The detection of these micrometastases by the OSNA assay could be explained either by the higher sensitivity of this method compared to conventional pathological ultrastaging or by “tissue allocation bias”, which has been extensively addressed in previous studies by our group and others [30,31]. Briefly, the random spatial distribution of metastases within the lymph node could result in their detection by different techniques being discordant. Unfortunately, in this type of comparative study between two techniques from a single sample (in this case, SLN), it is not possible to use exactly the same tissue for both tests. Especially in the case of low-volume metastases, there is the possibility that metastatic cells are located only in the part of tissue studied by OSNA and not in that analyzed by pathological ultrastaging. False-negative results (i.e., pathological ultrastaging positive/OSNA negative) were significantly less frequent; there were only 2/147 (1.3%) cases in which SLNs were negative by OSNA but positive by pathological ultrastaging, with a result of micrometastasis in both cases. In one of these cases, the OSNA assay detected the presence of ITCs, suggesting “tissue allocation bias” as a possible explanation of this discordance. Thus, the ability to analyze the whole lymph node (which diminishes the risk of sampling bias) allows the OSNA assay to detect small-volume metastases and ITC, demonstrating the greater sensitivity of the molecular analysis compared with pathological ultrastaging.

Previous studies demonstrated that the OSNA assay can be used intraoperatively in breast cancer [23,24,43]. In a recent meta-analysis, OSNA appears to be a highly accurate tool for intraoperative assessment of SLN in endometrial cancer [35]. The results of the ENDO-OSNA study confirm its potential for intraoperative analysis of SLN in endometrial cancer.

Several limitations of this study must be acknowledged. First, the spatial distribution of metastasis within a lymph node and the protocol for processing the SLNs in the study makes “tissue allocation bias” unavoidable. Additionally, discordant results between both tests may result from genuine differences between the tissue samples and the methods used for detecting metastases. Second, lack of follow-up means we do not know the recurrence rate among patients with metastatic burden detected by OSNA alone. Third, complete pathological ultrastaging (serial H&E + IHC) was performed in only 147 out of 191 recruited patients due to differences in sample processing procedures between participating centers. A hypothetical limitation of the study is the inability of OSNA to detect other biomarkers besides CK19, but several studies have demonstrated that CK19 mRNA is the best performing single marker for detecting lymph node metastasis in breast, lung, colorectal, prostate, cervical, and gastric cancers [23,24,25,26,27,28,29]. In this sense, we and others have demonstrated the reliability of CK19 mRNA in the detection of SLN metastasis in endometrial cancer [20,30,31,32,33,34].

## 5. Conclusions

OSNA is a sensitive and efficient alternative to pathological ultrastaging for the assessment of SLNB in EC. This molecular assay provides fast and reliable results and has already been successfully incorporated in the standard treatment guidelines for other tumors, such as breast cancer. In EC, the OSNA method shows higher sensitivity, specificity, and diagnostic accuracy in the detection of SLN metastasis, including low-volume metastasis, compared to pathological ultrastaging. OSNA also diminishes the risk of sampling bias since the whole lymph node is included for molecular analysis. The results of this study further demonstrate that OSNA can accurately detect SLN metastasis in early-stage EC patients and may help clinicians to provide the most appropriate treatment in each case.

## Figures and Tables

**Table 1:** General patient characteristics.

Characteristics	Patients (%)
Age at diagnosis (years)	
Average (SD)	63 (10.1)
Median (range)	62 (34–85)
Tumor Size (mm)	
Average (SD)	28.2 (16)
Median (range)	30 (1–95)
Histology	
Endometrioid	158 (82.7)
Serous	16 (8.4)
Carcinosarcoma	5 (2.6)
Clear cell	5 (2.6)
Mixed	3 (1.6)
Mucinous	2 (1.0)
Other	2 (1.1)
Tumor grade	
G1	85 (44.5)
G2	59 (30.9)
G3	41 (21.5)
Unknown	6 (3.1)
Myometrial invasion	
<50%	90 (47.1)
≥50%	63 (36)
No	22 (11.5)
Unknown	16 (8.4)
LVSI	
Yes	61 (32)
No	129 (67.5)
Unknown	1 (0.5)
Pelvic lymphadenectomy	
Yes	118 (61.8%)
Metastatic lymph nodes	12 (10.2%)
No	73 (38.2%)
Para-aortic lymphadenectomy	
Yes	62 (32.5%)
Metastatic lymph nodes	12 (19.2%)
No	129 (67.5%)

LVSI, lymphovascular space invasion.

**Table 2:** Summary of sentinel lymph node characteristics.

Sentinel Lymph Nodes (*n* = 526)	
Average/patient (SD)	2.8 (1.6)
Median (range)	2 (1–10)
Sentinel Lymph Node Size (mm)	
Average (SD)	14.7 (15.5)
Median (range)	12 (1.5–180)
Sentinel Lymph Node Weight (mg)	
Average (SD)	312.7 (345.4)
Median (range)	200 (0–2850)
OSNA (CK19 mRNA copies/µL)	
Average (SD)	1310 (10,155.5)
Median (range)	0 (0–156,000)

**Table 3:** Consistency between pathological ultrastaging analysis and OSNA.

	Pathological Ultrastaging		
	Positive	Negative	Total
**OSNA assay**			
**Positive**	11 (7.5%)	18 (12.2%)	29 (19.7%)
**Negative**	2 (1.4%)	116 (78.9%)	118 (80.3%)
**Total**	13 (8.8%)	134 (91.2%)	147 (100%)

**Table 4:** Summary of discordant cases between the OSNA assay and pathological ultrastaging.

No.	Age	Histology	Tumor (mm)	Grade	OSNA	CK19 mRNA (Copies/µL)	Ultrastaging	P-LA	PA-LA
1	61	Endometrioid	15	G2	(+)	3500	Negative	0/20	NP
2	56	Serous	1	G3	(+)	1500	Negative	1/17	0/12
3	60	Endometrioid	5	G3	(++)	5620	Negative	2/14	3/19
4	61	Endometrioid	15	G1	(+)	1270	Negative	0/7	0/5
5	71	Endometrioid	30	G1	(+)	270	Negative	0/4	0/1
6	74	Endometrioid	50	G2	(−)L	120	Micromet	0/23	NP
7	77	Endometrioid	40	G2	(++)	18,950	Negative	0/9	0/24
8	53	Endometrioid	42	G1	(+)	3100	Negative	NP	NP
9	52	Endometrioid	26	G1	(+)	410	Negative	NP	NP
10	57	Endometrioid	33	G1	(−)	0	Micromet	0/16	0/7
11	51	Endometrioid	40	G1	(+)	560	Negative	0/6	0/15
12	34	Endometrioid	15	G1	(+)	1630	Negative	NP	NP
13	75	Endometrioid	35	G2	(+)	1600	Negative	0/7	NP
14	68	Clear cell	NA	G3	(+)	1100	Negative	NP	NP
15	59	Endometrioid	23	G1	(+)	3100	Negative	0/11	NP
16	69	Endometrioid	7	G2	(+)	300	Negative	0/15	NP
17	48	Endometrioid	50	G1	(+)	530	Negative	0/11	NP
18	67	Endometrioid	30	G1	(+)	1580	Negative	0/5	NP
19	57	Clear cell	40	G3	(+)	470	Negative	NP	NP
20	59	Endometrioid	32	G1	(+)	960	Negative	0/2	NP

OSNA (qualitative result): (−), negative; (−)L, isolated tumor cells; (+), micrometastasis; (++), macrometastasis; P-LA, Pelvic lymphadenectomy; PA-LA, Para-aortic lymphadenectomy; NA, Not available; NP, Not performed.

**Table 5:** Changes in the TNM and FIGO staging according to the results of the OSNA assay.

					Ultrastaging		OSNA Staging	
Case	Age	Histology	Grade	LVSI	TNM	FIGO	TNM	FIGO
1	61	Endometrioid	G2	No	T1bN0M0	IB	T1bN1(mi)M0	IIIC1
5	71	Endometrioid	G1	No	T1aN0M0	IA	T1aN1(mi)M0	IIIC1
7	77	Endometrioid	G2	No	T1aN0M0	IA	T1aN1M0	IIIC1
11	51	Endometrioid	G1	No	T1bN0M0	IB	T1bN1(mi)M0	IIIC1
13	75	Endometrioid	G2	No	T1aN0M0	lA	T1bN1(mi)M0	IIIC1
14	68	Clear Cell	G3	No	T1bN0M0	lA	T1bN1(mi)M0	IIIC1
15	59	Endometrioid	G1	No	T1bN0M0	IB	T1bN1(mi)M0	IIIC1
16	69	Endometrioid	G2	No	T1bN0M0	IB	T1bN1(mi)M0	IIIC1
17	48	Endometrioid	G1	Yes	T1aN0M0	IA	T1aN1(mi)M0	IIIC1
18	67	Endometrioid	G1	Yes	T1aN0M0	lA	T1aN1(mi)M0	IIIC1
19	57	Clear Cell	G3	Yes	T2aN0M0	II	T2bN1(mi)M0	IIIC1
20	59	Endometrioid	G1	No	T1aN0M0	lA	T1bN1(mi)M0	IIIC1

**Table 6:** Lymphadenectomy results according to ultrastaging and OSNA: summary of discordant cases.

	OSNA-Positive/Ultrastaging-Negative	OSNA-Negative/Ultrastaging-Positive
Pelvic lymphadenectomy		
Positive	2 (15.4%)	0 (0.0%)
Negative	11 (84.6%)	2 (100.0%)
Para-aortic lymphadenectomy		
Positive	1 (16.7%)	0 (0.0%)
Negative	5 (83.3%)	1 (100.0%)

## Data Availability

The data presented in this study are not openly available due to confidentiality reasons but are available upon reasonable request from the corresponding authors.
